# High-throughput production of LuAG-based highly luminescent thick film scintillators for radiation detection and imaging

**DOI:** 10.1038/s41598-022-23839-w

**Published:** 2022-11-11

**Authors:** Shogen Matsumoto, Akihiko Ito

**Affiliations:** Graduate School of Environment and Information Sciences, Yokohama National University, 79-7, Tokiwadai, Hodogaya-Ku, Yokohama, 240-8501 Japan

**Keywords:** Materials for optics, Optical materials and structures

## Abstract

Radiography is non-destructive imaging for engineering, medical diagnostics, airport security checks, and decontamination activities in nuclear plants. Inorganic scintillators are phosphor materials that convert radiation into visible photons with high luminescence and fast response, and scintillators with a few tens of micrometers thickness can improve sensitivity in radiation detection and imaging. To date, a production method for thick film scintillators is a time and cost consuming way of slicing and poshing bulk single crystals and transparent ceramics. Here, the chemically vapor deposited Ce^3+^-doped Lu_3_Al_5_O_12_ thick film scintillators (CVD-Ce^3+^:LuAG) with a thickness of 1–25 μm were produced at deposition time of 1–30 min. Numerical simulations indicated the penetration depth of α-particle in Ce^3+^:LuAG is 12.8 μm, and the 14-μm-thick CVD-Ce^3+^:LuAG showed highest light yield (31,000 photons 5.5 MeV^−1^), superior to the commercial Ce^3+^:LuAG single crystal scintillator (21,000 photons 5.5 MeV^−1^). In the X-ray radiograph taken with CVD-Ce^3+^:LuAG as a scintillation screen, 5-μm-width bar of metal microgrids can be identified. Vapor deposition technique can be a novel high-throughput production way of a thick film scintillator which is in a micrometer-thickness effective to converting radiations into photons for sensitive α-emitter detection and high-resolution X-ray imaging.

## Introduction

X-ray imaging finds hidden contamination and damage for manufacturing engineering, medical diagnostics, and airport security controls, whereas α-particle imaging detects hazardous α-emitters in decontamination activities of nuclear plants^1–3^. Although powder scintillator screens have been used for a long time for their excellent light yields, their energy resolution is low due to their low opacity^4,5^. Conventional bulk scintillators of inorganic single crystal or ceramic with millimeter thickness can convert more radiation into visible photons due to the larger thickness. However, soft X-ray and α-particle generate radioluminescence in the range of several tens of micrometers from the surface of scintillation medium. In the bulk scintillators, light scattering and self-absorption reduce spatial resolution and light output in radiation imaging, and additional radioluminescence caused by γ-ray background complicates fast and sensitive α-particle detection^6,7^. Therefore, thick film scintillators, which have a comparable thickness with penetration depths of α-particle and soft X-ray, are expected to combine high light yield and high sensitivity to these radiations.

Ce^3+^-doped Lu_3_Al_5_O_12_ (Ce^3+^:LuAG) is a promising inorganic scintillation material with high effective atomic number (61), high light yield (25,000 photons MeV^−1^), and fast decay constants (50–70 ns) for high-resolution X-ray imaging^8,9^ and sensitive detection of α-emitters^10,11^. Ce^3+^:LuAG thick film scintillator is often produced by slicing and polishing of Ce^3+^:LuAG single crystal or transparent polycrystalline ceramic. Since LuAG has a high melting point (2300 K) with high hardness and brittleness, melt-growth or sintering followed by careful mechanical processes cause large energy consumption and material loss.

Deposition of LuAG thick film scintillator can be an alternative to slicing LuAG bulk scintillators. Zorenko et al. prepared 10–50-μm-thick film scintillators composed of LuAG using the liquid phase epitaxy (LPE) method^12–16^. The light yield of the films was reduced almost by half due to Ba or Pb contaminations during flux. Although pulsed laser deposition was used to prepare LuAG thin film, it is not suitable for thick film scintillators because the deposition rate was only 0.3 μm h^−1^^17^.

Laser-assisted chemical vapor deposition (CVD) technique can rapidly produce oxide thick films, for example Y_2_O_3_ and Lu_2_O_3_ sesquioxide phosphors, at high deposition rates, up to 20–100 μm h^−1^ faster than physical vapor deposition and conventional thermal CVD methods^18–22^. The obtained films were transparent and were close to or comparable with single crystal. However, the preparation of complex oxide scintillators, such as *AB*O_3_-type perovskite and *A*_3_*B*_5_O_12_-type garnet compounds, has merely been reported^23^. In addition, literature includes no study on α-particle detection with CVD-thick film scintillators.

In the present study, we proved a high-throughput production of LuAG-based thick film scintillators with CVD technique, and optimal thickness for α-particle detection was investigated with numerical simulations and scintillation spectrometry. Ce^3+^:LuAG thick film scintillators with a thickness up to 25 μm can be prepared within 30 min. Monte Carlo simulations and pulse-height spectrometry confirmed 14 μm-thickness is optimal for high sensitivity to α-particle detection (31,000 photons 5.5 MeV^−1^). X-ray imaging tests were performed on commercially available semiconductor storage card and metal microgrids. A high-resolution X-ray imaging was demonstrated by distinguishing 5-μm-width bar of metal microgrids in the radiograph taken with Ce^3+^:LuAG thick film scintillator.

## Results and discussion

### Estimation of optimal thickness of LuAG thick film scintillator for radiation detection

**Figure 1:**
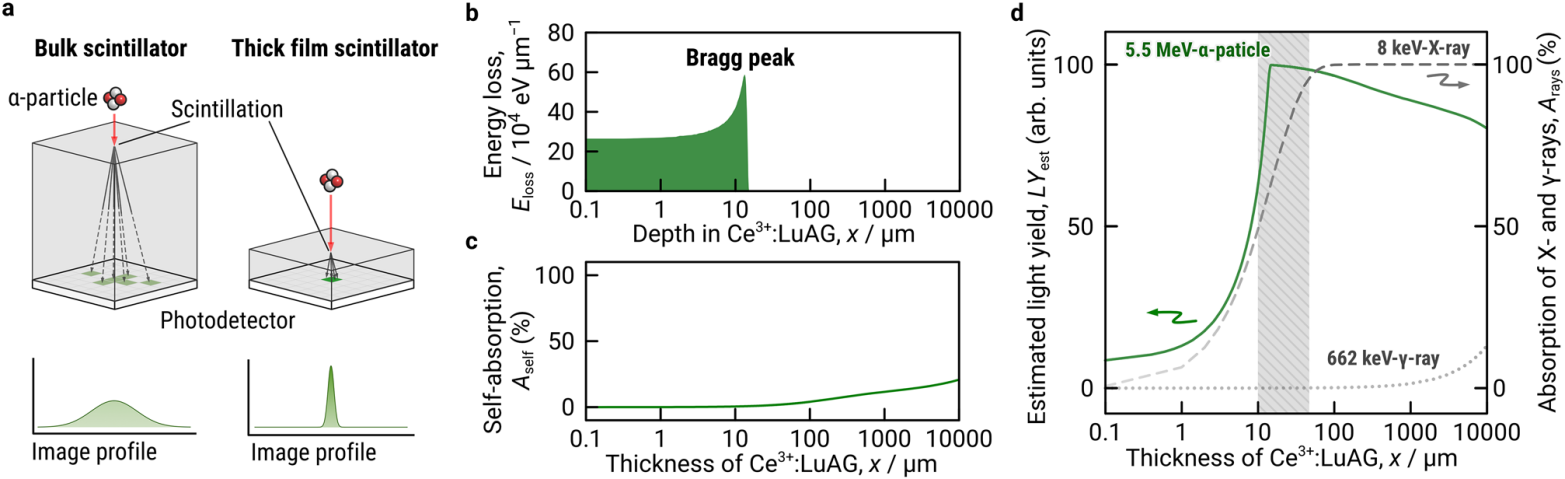
Estimation of optimal thickness of Ce^3+^:LuAG thick film scintillator for radiation detection. (**a**) A schematic representation of bulk and thick film scintillators coupled with photodetector for α-ray imaging (upper) and expected line profiles (lower). Dashed lines illustrate the light pass of scintillation light. (**b**) SRIM simulation of the energy loss spectrum of 5.5 MeV α-particle traveled in Ce^3+^:LuAG scintillator. (**c**) Calculated self-absorption of Ce^3+^:LuAG scintillator as a function of thickness of the Ce^3+^:LuAG scintillator for 5.5 MeV α-particle-induced radioluminescence of the Ce^3+^:LuAG scintillator. (**d**) Estimated light yield of Ce^3+^:LuAG scintillator for 5.5 MeV α-particle irradiation (solid line, left axis) and calculated absorption of 8 keV X-ray (dashed line, right axis) and 662 keV γ-ray (dotted line, right axis) in the Ce^3+^:LuAG scintillator. Hatched area indicates optimal thickness for α-particle and soft X-ray detections. The mass attenuation coefficients of LuAG for the irradiation of 8 keV X-ray and 662 keV γ-ray were retrieved from XCOM: Photon Cross Section Database (version 1.5). [Online] Available: http://physics.nist.gov/xcom.

Figure 1A shows a typical radiation detector in which inorganic scintillators are coupled with photodetectors such as complementary metal-oxide-semiconductor (CMOS) or photomultiplier (PMT) and trajectory of radioluminescence light in the scintillation media for α-particle irradiation. Bulk scintillators millimeter-thick single crystals and transparent polycrystalline ceramics have disadvantage of light scattering and self-absorption, which degrades spatial resolution and light output for radiation imaging. Among the radiations, α-particle has a penetration depth in micrometer scale, and thus absorption and scattering of radioluminescence light in scintillator medium is remarkable for the bulk scintillators^10^. Thus, there is an optimal thickness that obtains a high light yield produced by each radiation.

Figure 1b depicts the energy loss of 5.5 MeV^−1^ α-particle (*E*_loss_) in Ce^3+^:LuAG medium simulated by a Monte Carlo method using SRIM (Stopping and Range of Ions in Matter) and XCOM software packages^24–26^. A clear peak of energy loss, so-called Bragg peak, was observed at a depth of 12.8 μm. This indicates that Ce^3+^:LuAG thick film scintillator with such thickness can block α-particles completely. Some of the lost energy will be emitted as photons through ionization of the medium.

The effect of the thickness on self-absorption in Ce^3+^:LuAG medium was calculated using the Lambert–Beer equation (Eq. 1):

1 $${I}_{\mathrm{e}}= \sum {e}^{-\alpha \left(\uplambda \right) x I(\uplambda )},$$

where absorption coefficient (*α*(λ)) and emission intensity (*I*(λ)) were measured from a commercial Ce^3+^:LuAG single crystal at the wavelengths between 200 and 700 nm (Supplementary Fig. S1). The self-absorption became remarkable when the thickness of Ce^3+^:LuAG medium is more than 30 μm (Fig. 1c).

We estimated the light yield of Ce^3+^:LuAG for α-particle irradiation (*LY*_est_) as a function of Ce^3+^:LuAG thickness, taking into account *E*_loss_ and absorbance *A*_self_ (solid line in Fig. 1d). The estimated light yield increased exponentially with increasing the thickness and reached a maximum at the Bragg-peak depth of 12.8 μm, and then it gradually decreased due to self-absorption of the Ce^3+^:LuAG medium itself. Ce^3+^:LuAG is almost insensitive to 662 keV γ-ray up to thicknesses of 1000 μm (dotted line in Fig. 1d), indicating that LuAG with a 10–50-μm thickness can be α-particle detector for decontamination activities in nuclear plants by discriminating 5.5 MeV^−1^ α-particle radiation of PuO_2_ from γ-ray backgrounds. Furthermore, the thickness of 10–50 μm is consistent with the mean free path of soft X-ray (dashed line in Fig. 1d), indicating that these films have high detection sensitivity even for soft X-rays.

### CVD of Ce^3+^:LuAG thick film scintillator

**Figure 2:**
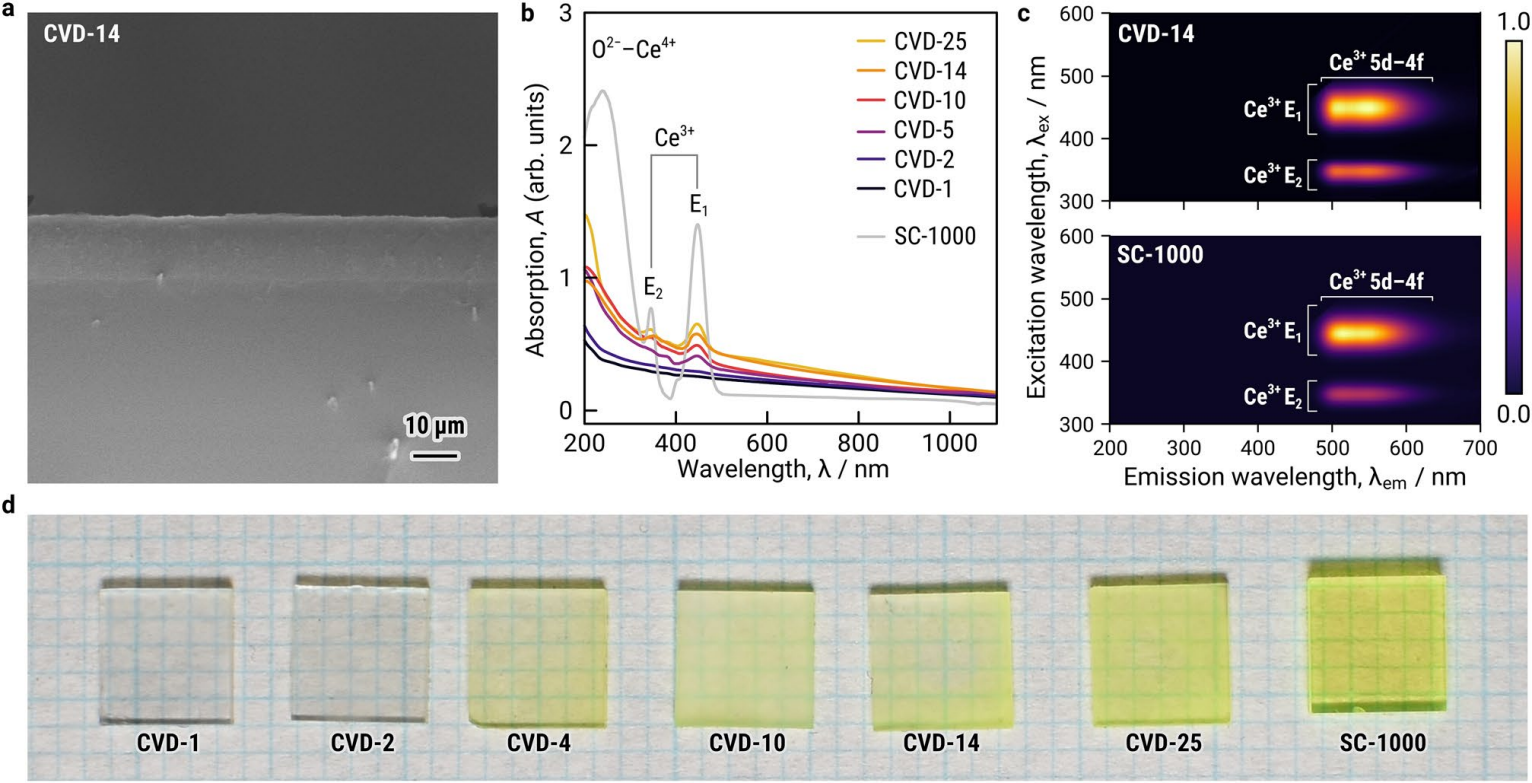
Characterization of CVD-Ce^3+^:LuAG thick film scintillators. (**a**) Cross-sectional SEM image of 14 μm-thick Ce^3+^:LuAG thick film scintillator grown on (100) YAG substrate (CVD-14). (**b**) Absorption spectra of the CVD-Ce^3+^:LuAG thick film scintillators with various thicknesses (CVD-1 to CVD-30) and reference Ce^3+^:LuAG single crystal (SC-1000). (**c**) Photoluminescence–excitation contour plot of CVD-20 and SC-1000. (**d**) Photographs of the Ce^3+^:LuAG thick film scintillators with various thicknesses (CVD-1 to CVD-30) and SC-1000 under room light.

Ce^3+^:LuAG thick films were epitaxially grown on Y_3_Al_5_O_12_ (YAG) single-crystal substrates by using CVD method. Out-of- and in-plane orientation relationship between the film and substrate was confirmed to be (100) LuAG || (100) YAG and [001] LuAG || [001] YAG (Supplementary Fig. S2). The thickness of the film was 14 μm for deposition time of 20 min (Fig. 2a). The thicknesses of the produced films were measured as 1, 2, 4, 10, 14 and 25 μm for deposition times of 1, 2, 5, 10, 20 and 30 min, as summarized in Supplementary Table S1, hereafter referred to as CVD-1 to CVD-25. For comparison, a commercially available Ce^3+^:LuAG single crystal with 1000 μm thickness and mechanically polished flake of Ce^3+^:LuAG with 30 μm thickness adhered on fused quartz substrate were used (hereafter referred to as SC-1000 and SC-30, respectively).

Optical absorption spectra of CVD-14 to CVD-25 and SC-1000 show absorption bands at 380 and 430 nm wavelengths, which are attributed to the E_1_ and E_2_ levels of the Ce^3+^ center (Fig. 2b). The level of absorption derived from the Ce^3+^ centers increased with the increasing thickness of films, following the Lambert–Beer law. Figure 2c shows the photoluminescence (PL)–excitation (PLE) contour plots of CVD-14 and SC-1000. In both CVD-14 and SC-1000, emission from the 5d–4f transition of Ce^3+^ was observed at wavelengths of 480–640 nm. Figure 2d shows the photographs of the CVD specimens. Yellowish color agrees with the observed absorption bands, and the color became stronger as the thickness of the Ce^3+^:LuAG thick film increased.

### α-Particle detection with CVD-Ce^3+^:LuAG

**Figure 3:**
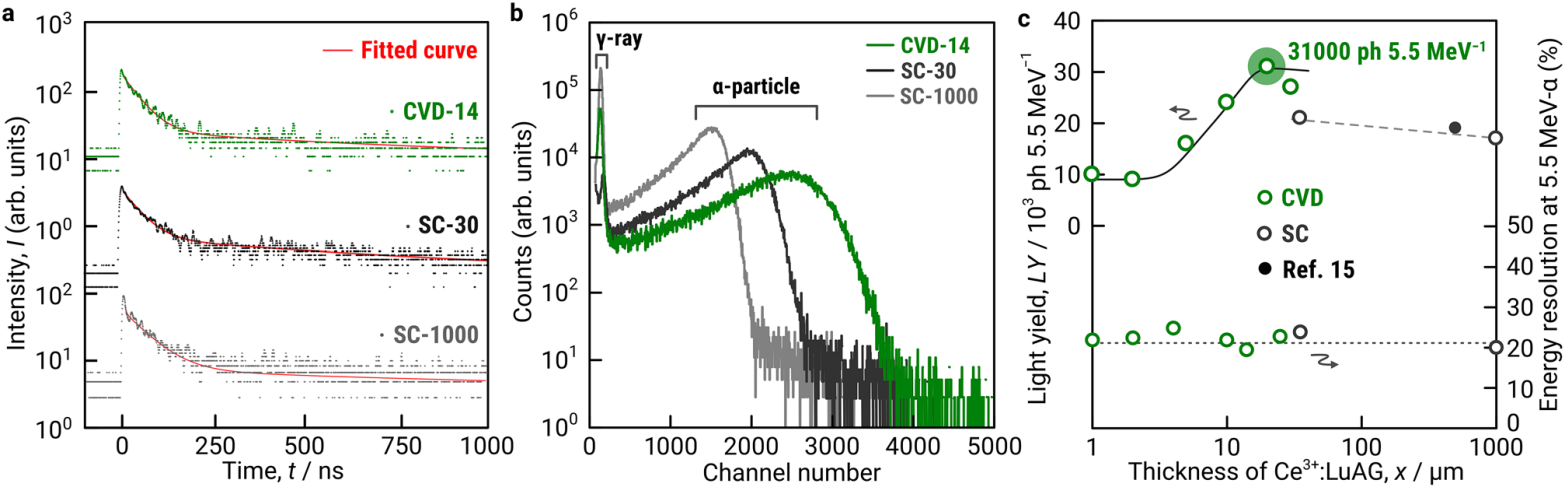
α-Particle-induced scintillation properties of the Ce^3+^:LuAG thick film scintillators. (**a**) α-Particle-induced decay profile of CVD-14, SC-30, and SC-1000. Solid lines indicate fitted curves of two exponential functions. (**b**) Pulse height spectra of CVD-14, SC-30, and SC-1000. (**c**) Light yield and energy resolution of the Ce^3+^:LuAG thick film scintillators (CVD-1 to CVD-25) as a function of its thickness and those of reference Ce^3+^:LuAG single crystals (SC-30, SC-1000, and ref. 15).

^241^Am was used as a 5.5-MeV α-particle source for the evaluation of scintillation decay and light yield of Ce^3+^:LuAG thick film scintillators. The measured decay curve was fitted by two exponential functions with fast and slow decay components (τ_1_ and τ_2_, respectively). The τ_1_ and τ_2_ of CVD-14 were 39 and 594 ns, respectively (Fig. 3a). These values were comparable with SC-1000 and SC-30 (Supplementary Fig. S3 and Table S1). The fast and slow decay constant was derived from the 5d–4f transition of the Ce^3+^ center and antisite defect (Lu_Al_), respectively^23^. The yellow-green light emitted from the Ce^3+^ center was also observed by α-particle-induced luminescence spectra for CVD-14, SC-30, and SC-1000, which were in agreement with ultraviolet (UV)-induced luminescence spectra (Supplementary Fig. S4).

In pulse height spectra measurements, total absorption peak of α-particle for CVD-14 appeared at a higher channel than those for SC-1000 and SC-30 (Fig. 3b). The light yield of SC-1000 and SC-30 was calculated as 21,000 and 17,000 photons 5.5 MeV^−1^, respectively, and these values were similar to the reported value of 500-μm-thick Ce^3+^:LuAG sliced from bulk single crystal (19,000 photon 5.5 MeV^−1^)^15^. The light yield of CVD-14 is calculated as 31,000 photon 5.5 MeV^−1^ and is higher than those of SC-30 and SC-1000. In the literature, the light yield of LPE-grown 10–50-μm-thick Ce^3+^:LuAG-based thick film scintillator has been reported as 10,000–14,000 photons 5.5 MeV^−1^, and these low values were associated with Ba or Pb contaminations from BaO and PbO fluxes used in the LPE process^15,16^. The CVD process can avoid contaminations of transition metal elements, which significantly reduces the light yield of radioluminescence.

We summarized the effect of thickness on the light yield of Ce^3+^:LuAG thick film scintillators (Fig. 3c). The light yield of the Ce^3+^:LuAG thick film scintillator increased from 9000 to 31,000 photons 5.5 MeV^−1^ as the film thickness increased from 1 to 14 μm, and it decreased to 27,000 photons 5.5 MeV^−1^ for 25 μm thickness. This trend is in good agreement with the prediction made with SRIM simulation as discussed in the above section. The optimal thickness of 14 μm for α-particle detection was confirmed by both simulation and experimental results. The energy resolutions of both CVD and SC specimens were 20.1–25.4%, and there is no remarkable difference between CVD and SC specimens (Fig. 3c).

### High-resolution X-ray imaging test with CVD-Ce^***3***+^***:LuAG***

**Figure 4:**
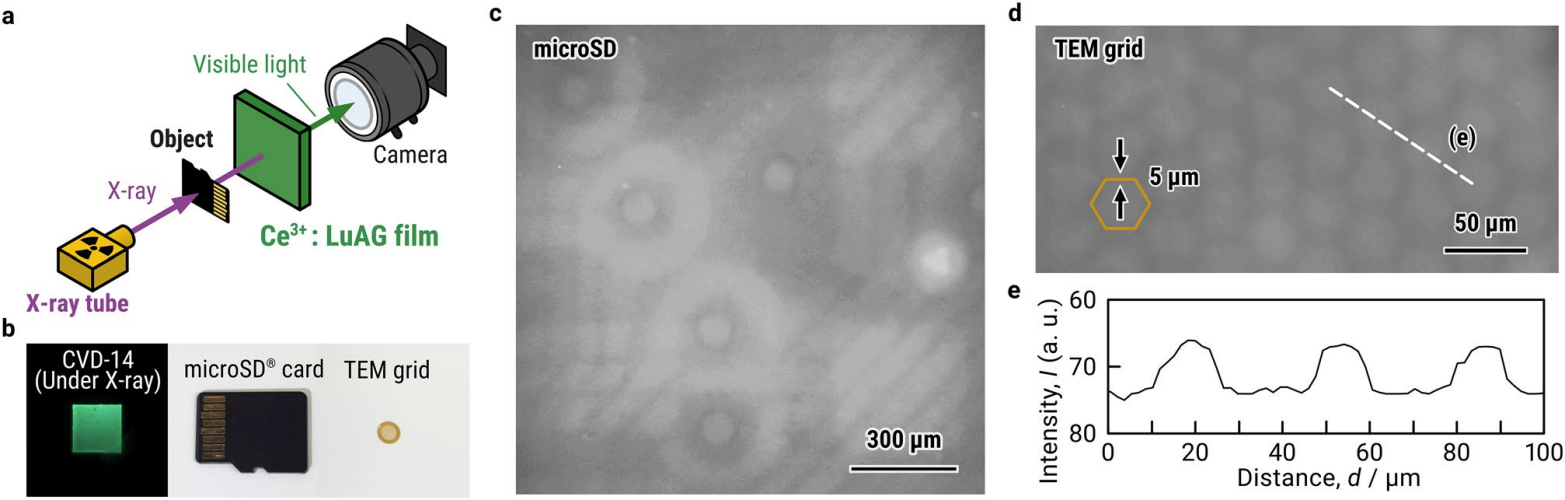
High-resolution X-ray imaging test. (**a**) A schematic of high-resolution X-ray imaging test using CVD-14. (**b**) Photograph of CVD-14 under X-ray irradiation as a scintillation screen (left), and radiographic subjects of microSD card (center) and TEM grid (right) under room light. (**c**) X-ray radiograph of microSD card. (**d**) X-ray radiograph of TEM grid. Solid line depicts a schematic of hexagonal mesh made with 5 μm-width bar. (**e**) Line intensity profile along the dashed line in the radiograph (**d**).

CVD-14 was used to perform high-resolution X-ray imaging test of microSD card and TEM grid with commercially available X-ray tube and CCD camera (Fig. 4a,b). CVD-14 emitted a green radioluminescence at a wavelength of 540 nm under X-ray irradiation (Fig. 4b and Supplementary Fig. S5). X-ray afterglow response of CVD-14 was the same compared with SC-30. Through-holes inside the semiconductor storage card and hexagonal mesh of the TEM grid are visualized as shown in Figs. 4c,d. The line intensity profile along dashed line in Fig. 4d confirmed that we can identity 5 μm-width bar of hexagonal mesh in the radiograph taken with CVD-14 (Fig. 4e) and CVD-thick film scintillators are suitable for high-resolution X-ray imaging.

In summary, we demonstrated the high-throughput production of LuAG-based highly luminescent thick film scintillators for radiation detection and imaging. The Ce^3+^:LuAG thick film scintillators with a thickness up to 25 μm can be prepared within 30 min. Monte Carlo simulations and Lambert–Beer raw indicated the light yield increased exponentially with increasing the Ce^3+^:LuAG thickness and reached a maximum at the Bragg-peak depth of 12.8 μm, and then it gradually decreased due to self-absorption of the Ce^3+^:LuAG medium itself. The light yield of the Ce^3+^:LuAG thick film scintillator increased from 9000 to 31,000 photons 5.5 MeV^−1^ as the film thickness increased from 1 to 14 μm, and it decreased to 27,000 photons 5.5 MeV^−1^ for 25 μm thickness. This trend was in good agreement with the estimation. The highest light yield of 31,000 photon 5.5 MeV^−1^ was observed for 14 μm-thick Ce^3+^:LuAG thick film scintillator (CVD-14) and is higher than those of SC-30 and SC-1000 (21,000 and 17,000 photons 5.5 MeV^−1^, respectively). This is because α-particle has a penetration depth in micrometer scale and self-absorption and scattering of radioluminescence light in scintillator medium is remarkable for the bulk scintillators. The 14 μm-thick CVD-Ce^3+^:LuAG thick film scintillator was used as a scintillation screen to perform high-resolution X-ray imaging test of microSD card and TEM grid. 5 μm-spatial resolution can be confirmed in the radiographs. It has been believed that slicing and polishing of single crystals or polycrystalline transparent ceramics is the only way to produce thick film scintillators. We proved the concept that thick film scintillators are highly luminescent phosphors with low self-absorption for high-sensitivity detection of α-particles and high-resolution X-ray imaging, and synthesizing thick film scintillators by using vapor deposition method saves time and cost compared to slicing single crystals and polycrystalline transparent ceramics.

## Materials and methods

### Preparation of Ce^3+^:LuAG scintillator films

The laser-assisted CVD apparatus used in this study has been reported in a previous study^27^. Metal–organic precursors of lutetium tris (dipivaloylmethanate) (Strem Chemicals, USA), aluminum tris (acetylacetonate) (Merck, USA), and cerium tetrakis (dipivaloylmethanate) (Toshima Manufacturing, Japan) were maintained at temperatures of 453, 453 and 493 K, respectively. The resultant vapors were introduced into the deposition chamber using Ar as a carrier gas, and O_2_ gas was separately introduced into the chamber through a double-tube nozzle; the total chamber pressure was kept at 0.2 kPa. The molar ratio of Ce:Lu:Al = 1:33:66 (3–4 mol%Ce) in the precursor vapor was calculated from the mass change in each precursor before and after deposition. The YAG single crystal (5 × 5 × 0.5 mm^3^, both sides polished) was used as a substrate and heated at 1000 K with an electrical heating stage, and then it was irradiated with a CO_2_ laser (wavelength: 10.6 μm; maximum laser output: 60 W; SPT Laser Technology, China) through a ZnSe window. The deposition temperature was 1193 K under laser irradiation, and the deposition time was conducted at 1–30 min. The yield of CVD-25 thick film scintillator estimated from the amount of raw material consumed was 4.1%.

### Phase composition, microstructure and optical properties

The phase composition of the resultant film was identified by θ–2θ XRD (Bruker D2 Phaser, USA), and the in-plane orientation was evaluated by ϕ-scan XRD measurements (Rigaku Ultima IV, Japan). The optical absorption and photoluminescence spectra were measured using a UV–visible spectrophotometer (JASCO V-630, Japan) and a fluorescence spectrophotometer (JASCO FP-8300, Japan), respectively. The microstructure and thickness of the films was observed using a scanning electron microscope (Hitachi SU-8010, Japan).

### α-Particle-induced scintillation spectrometry

^241^Am source was used as a 5.5-MeV α-particle and 662 keV γ-ray source. α-Particle-induced luminescence spectra were recorded using a spectrophotometer (JASCO FP-8300, Japan) with the PMT spectral correction sensitivity. The scintillation decay curve and pulse height spectra were measured using a PMT (Hamamatsu Photonics R7600-200, Japan). The specimens were mounted on the window of the PMT with a thin layer of optical silicone grease (OHYO KOKEN KOGYO TSK5353, Japan). The decay profile was recorded using an oscilloscope (IWATSU ELECTRIC DS-5622A, Japan). In pulse height spectra measurements, the signal was fed to a pre-amplifier (ORTEC Model 113, USA), shaping amplifier (ORTEC Model 572A, USA), and a multichannel analyzer (AMPTEK MCA-8000D, USA). The capacitance of the pre-amplifier and the shaping time of the shaping amplifier were set at 1000 pF and 2 μs, respectively. The 700 V bias for the PMT was supplied by an HV power supply (CAEN DT5471P, Italy). After the correction of PMT spectral sensitivity (see Supplementary Fig. S7), the light yield and energy resolution were calculated from the channel number corresponding to the center of the Gaussian peak and full width at half maximum (FWHM) of the Gaussian peak, respectively^28,29^. The scintillation properties of commercially available Ce^3+^:LuAG single crystals (0.2 mol%Ce, 25,000 photons MeV^−1^; Epic Crystal, China) were also measured in the same setup^30^.

### High-resolution X-ray imaging test

Commercially available X-ray source (Cu target operated at an acceleration voltage of 40 kV and applied current of 40 mA) was used to perform X-ray-induced scintillation spectrometry and X-ray imaging test. X-ray-induced scintillation spectra were measured with a multichannel spectrometer with a cooling mechanism (StellarNet SLIVER-Nova, USA). The X-ray afterglow and radiograph were obtained using the X-ray source and the CMOS camera (number of pixels: 1304 × 976 pixels; pixel size: 3.75 × 3.75 μm^2^; ZWO ASI224MC, China) with objective lens (magnification: 10× , OLYMPUS MPLFLN10X, Japan). After irradiating the specimen with X-rays for 2 s, the X-rays were shielded using a 2-mm thick Pb plate to obtain an afterglow curve. X-ray imaging tests were performed with microSD card and TEM grids made with Au hexagonal mesh (5 μm bar and 35 μm pitch).

## Supplementary Information

Supplementary Information.

## Data Availability

The datasets generated during and/or analyzed during the current study are available from the corresponding author on reasonable request.
