# Supplementary material for: High-throughput production of LuAG-based highly luminescent thick film scintillators for radiation detection and imaging
Source: Sci Rep. 2022 Nov 11;12:19319. doi: 10.1038/s41598-022-23839-w (PMC9652359; doi:10.1038/s41598-022-23839-w)
Supplement: Supplementary file 1 — Supplementary Information. [file 41598_2022_23839_MOESM1_ESM.pdf]

SUPPLEMENTALY INFORMATION

High-throughput Production of LuAG-based Highly Luminescent Thick Film Scintillators for Radiation Detection and Imaging

Shogen Matsumoto[1], *Akihiko Ito*[1,*]

[1]*Graduate School of Environment and Information Sciences, Yokohama National University, 79-7, Tokiwadai, Hodogaya-Ku, Yokohama 240-8501, Japan*

[*]*Corresponding author: E-mail: ito-akihiko-xr@ynu.ac.jp, Tel: +81-45-339-3354*

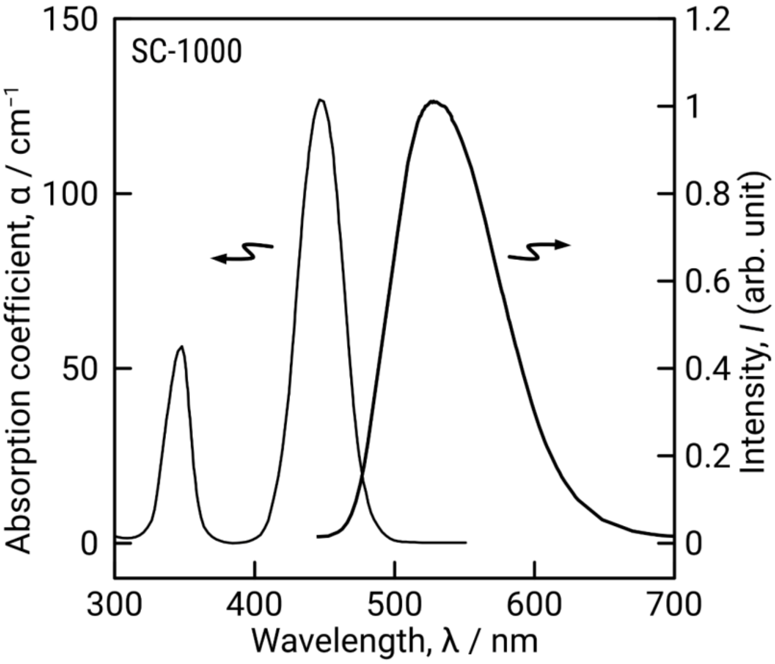

Supplementary Fig. S1 Optical absorption and photoluminescence spectra of Ce^{3+}:LuAG single crystal (SC-1000).

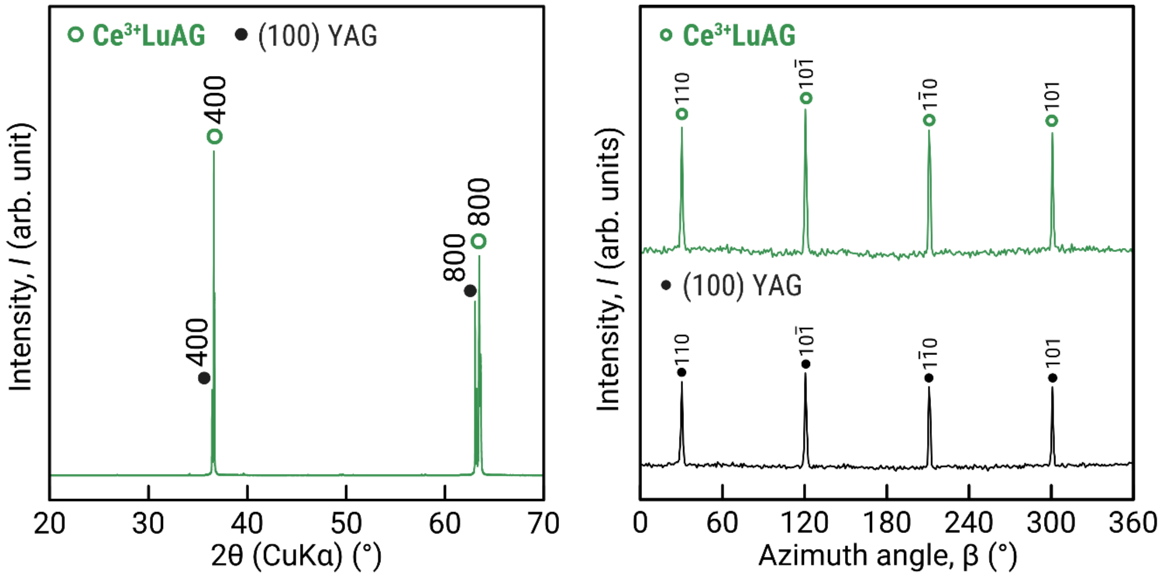

Supplementary Fig. S2 θ–2θ XRD pattern and ϕ-scan XRD patterns at LuAG {110} and YAG {110} planes.

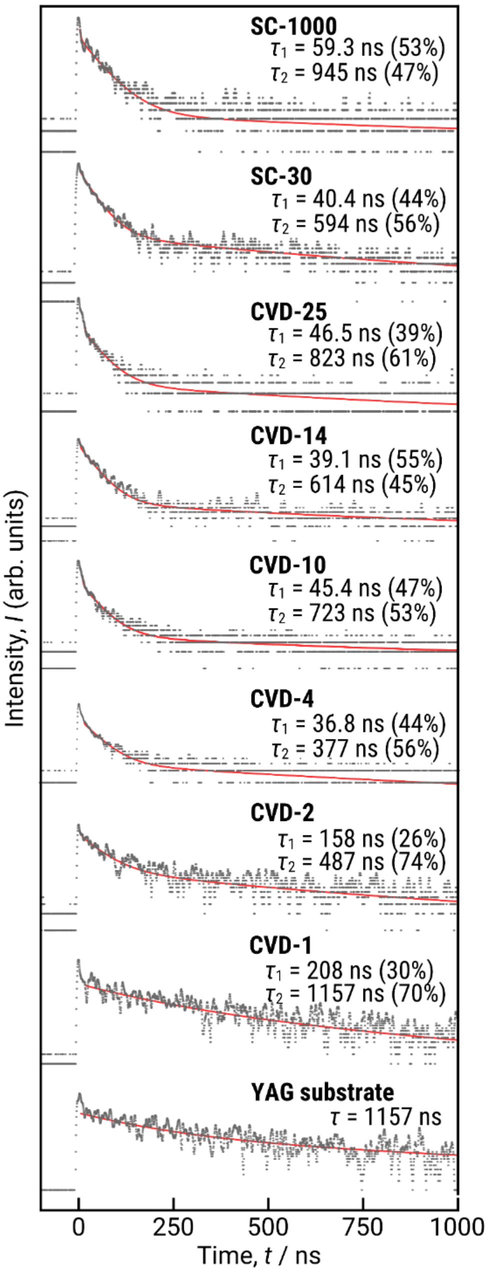

Supplementary Fig. S3 α-particle-induced scintillation decay profile of raw YAG substrate, CVD-1, CVD-2, CVD-4, CVD-10, CVD-14, CVD-25, SC-30, and SC-1000.

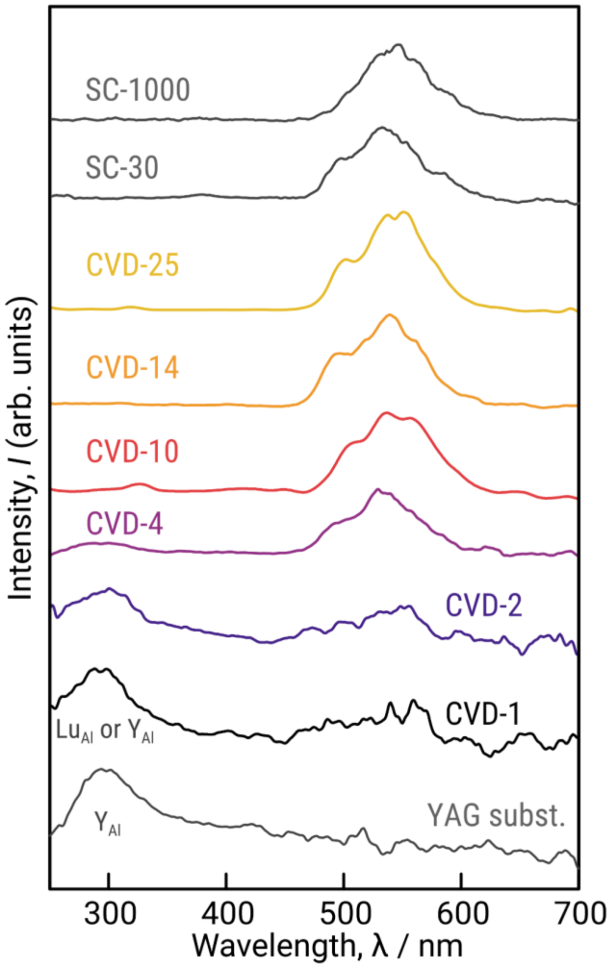

Supplementary Fig. S4 α-particle-induced scintillation spectra of raw YAG substrate, CVD-1, CVD-2, CVD-4, CVD-10, CVD-14, CVD-25, SC-30, and SC-1000.

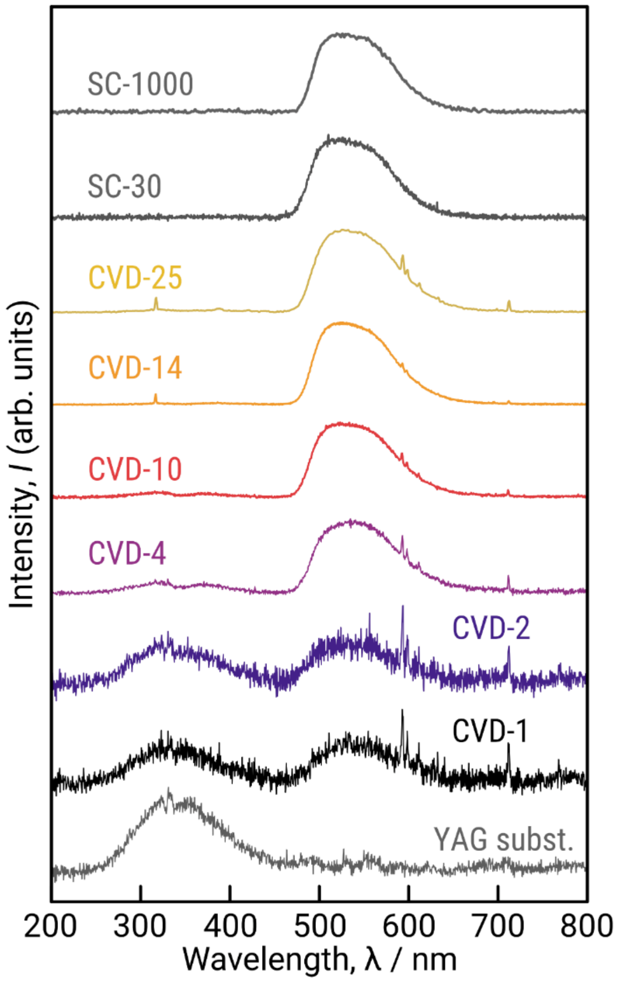

Supplementary Fig. S5 X-ray-induced scintillation spectra of raw YAG substrate, CVD-1, CVD-2, CVD-4, CVD-10, CVD-14, CVD-25, SC-30, and SC-1000.

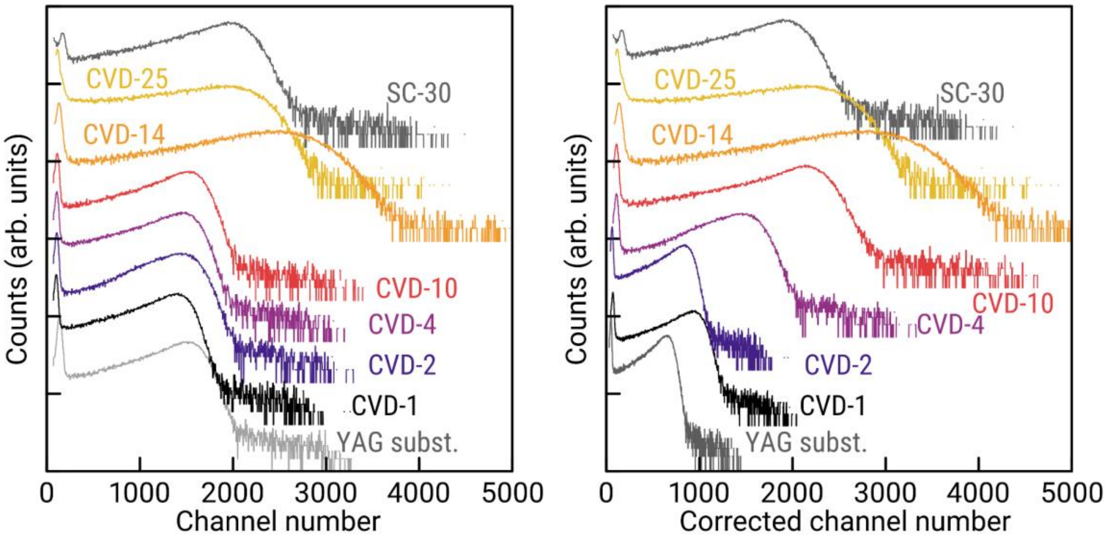

Supplementary Fig. S6 α-particle-induced pulse height measurements before and after correction for the PMT quantum efficiency of as-received YAG substrate, CVD-1, CVD-2, CVD-4, CVD-10, CVD-14, CVD-25, and SC-30. SC-30 was used as the standard for correction.

Table S1 Summary of thickness and scintillation properties of CVD-Ce^{3+}:LuAG thick film scintillators and LuAG single crystal.

Sample	Thickness / μm	Fast decay component / ns	Slow decay component / ns	Light yield / photons 5.5 MeV^{-1}	Energy resolution at 5.5 MeV-α-ray (%)
YAG subst.	-	1157	-	9000	20.3
CVD-1	0.8	208 (30%)	1157 (70%)	10000	22.3
CVD-2	1.8	158 (26%)	487 (74%)	9000	22.7
CVD-4	3.9	36.8 (44%)	377 (56%)	16000	25.4
CVD-10	10.0	45.4 (47%)	723 (53%)	24000	22.2
CVD-14	13.9	39.1 (55%)	614 (45%)	31000	20.1
CVD-25	24.7	46.5 (39%)	823 (61%)	27000	23.1
SC-30	34	40.4 (44%)	594 (56%)	21000	24.3
SC-1000	1000	59.3 (53%)	945 (47%)	17000	20.5
